# Frameshift peptides alter the properties of truncated FUS proteins in ALS-FUS

**DOI:** 10.1186/s13041-020-00618-0

**Published:** 2020-05-13

**Authors:** Haiyan An, Camille Rabesahala de Meritens, Vladimir L. Buchman, Tatyana A. Shelkovnikova

**Affiliations:** 1 Medicines Discovery Institute, Cardiff University, Cardiff, CF10 3AT UK; 2 Biomedicine Division, School of Biosciences, Cardiff University, Park Place, Cardiff, CF10 3AT UK

**Keywords:** Fused in sarcoma, FUS, Amyotrophic lateral sclerosis, ALS, Frameshift mutation, Frameshift peptide, Stress granule

## Abstract

Mutations in the *FUS* gene cause a subset of ALS cases (ALS-FUS). The majority of *FUS* mutations are missense mutations affecting the nuclear localisation signal (NLS) of FUS. In addition, a number of frameshift mutations which result in complete NLS deletion have been described. Patients bearing frameshift mutations usually present with more aggressive disease, characterised by an early onset and rapid progression. Both missense mutations in the NLS coding sequence and complete loss of the NLS are known to result in cytoplasmic mislocalisation of FUS protein. However, in addition to the removal of FUS functional domains, frameshift mutations in most cases lead to the attachment of a “tail” of novel amino acids at the FUS C-terminus – a frameshift peptide. It is not clear whether these peptide tails would affect the properties of truncated FUS proteins. In the current study, we compared intracellular behaviour of disease-associated truncated FUS proteins with and without the corresponding frameshift peptides. We demonstrate that some of these peptides can affect subcellular distribution and/or increase aggregation capacity and stability of the truncated FUS protein. Our study suggests that frameshift peptides can alter the properties of truncated FUS variants which may modulate FUS pathogenicity and contribute to the variability of the disease course in ALS-FUS.

## Main text

Mutations in the *FUS* gene are responsible for a subset of amyotrophic lateral sclerosis (ALS) cases referred to as ALS-FUS [1, 2]. In these cases, FUS protein is found accumulated in the cytoplasm of motor neurons and glial cells in a form of pathological inclusions, concomitant with its partial loss from the nucleus. The majority of mutations occur in the sequences encoding the nuclear localisation signal (NLS) in the C-terminal part of FUS molecule. Compromised function of the NLS results in deficient nuclear import and abnormal cytoplasmic accumulation of the protein. Most of FUS NLS-mapping mutations are missense mutations, however, other types of mutations such as indels, duplications and splicing site mutations have been also reported in familial and sporadic ALS cases [3, 4]. These latter types of mutations often cause a frameshift leading to the production of a truncated FUS protein lacking NLS and sometimes adjacent protein sequences but having a C-terminal “tail” of novel amino acids – a frameshift peptide. Patients bearing frameshift mutations display a more aggressive form of the disease characterised by an early disease onset, rapid progression and shorter life expectancy, compared to patients carrying missense mutations [3]. It is not clear whether the frameshift peptides can confer additional pathogenic characteristics to the truncated FUS protein. To address this, we constructed a panel of plasmids for the expression of ALS-linked truncated FUS proteins with or without respective frameshift peptide tails, and analysed their cellular distribution, levels and stability in human neuroblastoma cells.

**Fig. 1:**
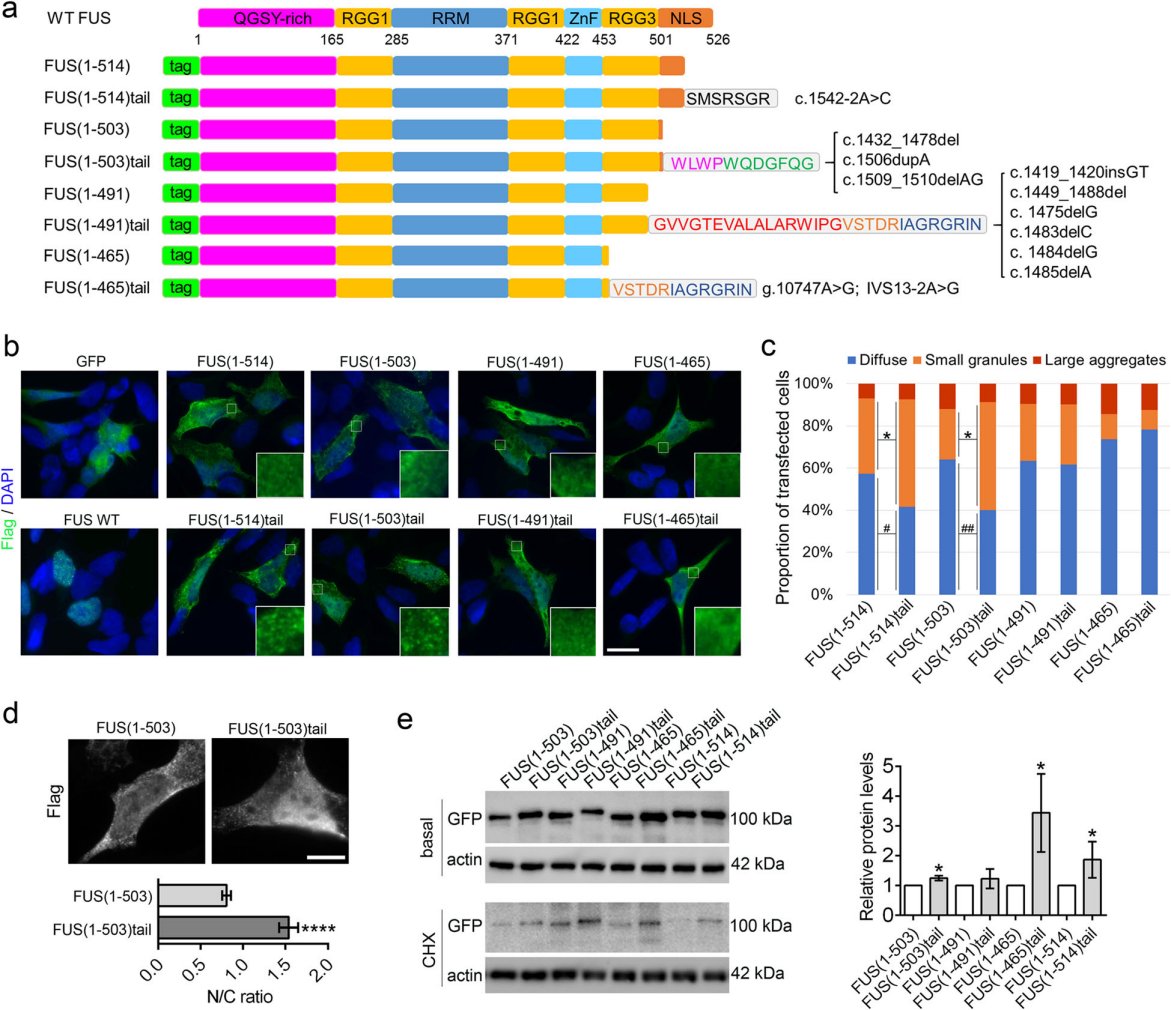
The effect of frameshift peptides on the properties of truncated FUS variants associated with ALS. **a** Diagrams showing FUS variants used in the study. Protein sequences of frameshift peptides attached to the respective FUS variant are also shown. Two types of tags were used, Flag and GFP. **b** Subcellular localisation of FUS variants with and without respective frameshift peptide tails when expressed as Flag fusions in SH-SY5Y cells. Cells were analysed 24 h post-transfection. Representative images are shown. Insets show fine granular aggregates (“small granules”) in the cytoplasm of cells expressing FUS(1–503)tail and FUS(1–514)tail variants. Scale bar, 10 μm. **c** Quantification of cytoplasmic aggregation of FUS variants with and without tail expressed as Flag fusions. Cells were analysed 24 h post-transfection. Between 166 and 201 cells were analysed per variant. * and ^#^ - *p* < 0.05, ^##^*p* < 0.01, FUS(1–514) vs. FUS(1–514)tail and FUS(1–503) vs. FUS(1–503)tail (Mann-Whitney *U* test). **d** Increased nuclear retention of FUS(1–503) variant conferred by the attachment of the respective frameshift peptide. Representative images and quantification of nuclear/cytoplasmic (N/C) FUS ratio are shown. Forty cells were analysed per variant. ****p < 0.0001 (Student’s *t* test). Scale bar, 5 μm. **e** Increased stability of FUS variants with frameshift tails. Cells were transfected to express GFP-tagged FUS proteins, and 24 h post-transfection, cycloheximide (CHX) was added for 36 h. Protein levels were analysed by western blot with an anti-GFP antibody and results were quantified by densitometry (*n* = 4). Protein levels in CHX-treated samples were normalised to basal protein levels. *p < 0.05 (Mann-Whitney *U* test)

Firstly, we systematically reviewed the existing literature to collect information of reported truncation mutations in the *FUS* gene. Altogether, we have found reports of 13 different frameshift mutations, with the majority being deletions (Additional file 1: Table S1) [5–12]. Notably, out of 16 cases with reported disease onset, all patients, except one, were diagnosed before reaching the age of 50 and usually in their 20’s. Mutant protein products in all these cases are predicted to have a C-terminal truncation and a frameshift peptide, with the peptide lengths varying between 7 and 55 amino acids. Certain amino sequences appear in more than one frameshift peptide tail (Additional file 1: Table S1), and four of these common sequences were selected for analysis (Fig. 1a). Constructs to express truncated FUS proteins with or without the corresponding tail, as N-terminal GFP or Flag tag fusions, were generated (for detailed methods, see Additional file 1). Frameshift peptides were attached to the corresponding truncated FUS variants yielding a panel of eight constructs which could be compared in a pairwise fashion: FUS(1–465), FUS(1–465)tail; FUS(1–491), FUS(1–491)tail; FUS(1–503), FUS(1–503)tail; FUS(1–514), FUS(1–514)tail (Fig. 1a). Three of these variants with frameshift tails were native isoforms predicted to occur in patients, namely, FUS(1–514)tail, FUS(1–503)tail and FUS(1–465)tail. In addition, we generated a construct to express a protein representative of a cluster of six truncation mutations affecting the distal (C-terminal) portion of the RGG3 domain (mutations truncating FUS from aa. 473 to aa. 496) and all sharing the common sequence “GVVGTEVALALARWIPGVSTDRIAGRGRIN” in their tails (Fig. 1a). The resultant engineered protein, FUS(1–491)tail, had an “intermediate”-length truncation and had the above common tail attached to it. Therefore, the use of these four types of mutants allowed us to cover four types of truncations in FUS protein: loss of the distal portion of the NLS (1–514); almost the entire NLS (1–503); the NLS/distal portion of RGG3 domain (1–491); and the NLS/almost the entire RGG3 domain (1–465), with three mutants being native ones. For analysis of protein distribution and aggregation in human SH-SY5Y cells, we used Flag-tagged versions which are expressed at lower, close to physiological levels, as compared to highly accumulated GFP-tagged ones [13].

Analysis of FUS protein distribution showed that all proteins were present in the cytoplasm, as compared to FUS WT which is exclusively nuclear, in agreement with the NLS loss/impairment and hence nuclear import defect (Fig. 1b). RGG3 box important for RNA binding is affected by longer FUS truncations, which may impact on the FUS properties requiring RNA binding, e.g. its incorporation into RNA granules. Mutant FUS is able to spontaneously form small RNA granules and their clusters in the cytoplasm [13]. Quantification of cells with such spontaneous small granules and their clusters (large aggregates) showed that FUS(1–465) mutant lacking almost the entire RGG3 had the lowest (26 ± 9%) while FUS(1–514) mutant with only the NLS affected had the highest (43 ± 5%) proportion of cells with cytoplasmic granules (Fig. 1c). Further, using sodium arsenite treatment to induce physiological RNA granules stress granules (SGs), we found that FUS(1–514) and FUS(1–503) variants demonstrated near-complete sequestration into SGs without diffuse protein remaining in the cytoplasm, whereas FUS(1–491) and FUS(1–465) variants presented with a significant amount of diffuse cytoplasmic FUS (Additional file 1: Figure S1). Therefore, the affinity of FUS to RNA granules is negatively affected by larger truncations which disrupt RGG3 domain.

Pairwise comparison of variants with and without frameshift peptide tail showed that the presence of any of the tails did not significantly alter the recruitment of truncated FUS into SGs. However, it revealed that some tails affect spontaneous cytoplasmic granule assembly and subcellular localisation of truncated FUS. Firstly, we detected augmented cytoplasmic granule formation in the presence of tails for two truncated proteins, FUS(1–514) and FUS(1–503) (Fig. 1b, c). Further, we found that attachment of the respective tail increased nuclear retention of FUS(1–503) variant whereas other frameshift peptides did not visibly affect subcellular FUS distribution (Fig. 1d). We next examined the stability of FUS proteins with and without peptide tail using cycloheximide (CHX) pulse chase. FUS proteins with and without tails were expressed at a similar basal level, and CHX treatment of neuroblastoma cells for 36 h led to reduced FUS protein levels (Fig. 1e). However, three out of four frameshift tails (all native ones) significantly increased the stability of FUS protein (Fig. 1e). In contrast, tagging GFP with any of the peptide tails studied did not increase its stability (Additional file 1: Figure S2) which indicates that the effect of the tail on the protein stability is realised in the context of FUS protein but not any generic protein.

Several conclusions can be made from the obtained experimental data. Firstly, we show that ALS-linked FUS truncations affecting RGG3 domain reduce RNA granule affinity of the protein and this effect is independent of the presence or absence of a frameshift peptide. Thus larger truncations are likely to lead to the loss of FUS function in RNA granule-regulated cellular processes. Secondly, we found that for truncations affecting the NLS but not RGG3 domain, some frameshift peptide tails can confer increased propensity to form cytoplasmic FUS-positive granules. Thirdly, we show that some frameshift peptides arising as a result of a deletion, duplication or splicing site mutation in the *FUS* gene can increase the stability of mutant FUS variants. Increased FUS protein stability coupled with enhanced propensity to form cytoplasmic granules may facilitate inclusion formation in the cytoplasm of affected neurons in patients. Finally, we found that one frameshift peptide, WLWPWQDGFQG, can increase nuclear retention of mutant FUS. On the one hand, presence of this peptide can partially rescue cytoplasmic FUS deposition, on the other hand, accumulation of mutant FUS in the nucleus can result in toxic gain of function [14]. Notably, some of the frameshift peptides, e.g. (VSTDR)IAGRGRIN, add novel arginine residues to the truncated mutants and therefore might modulate methylation patterns of the resultant mutant proteins. Methylation of arginine residues upstream FUS NLS is believed to regulate cytoplasmic accumulation and aggregation of mutant FUS protein [15].

In conclusion, in this study we demonstrate that individual frameshift peptides confer distinct properties to the truncated FUS proteins produced as a result of certain ALS-linked *FUS* gene mutations. Presence of these peptides adds another layer of complexity to the ALS-FUS molecular pathogenesis. Thus, studies of this highly heterogeneous class of mutations should be based upon natural FUS variants comprising the respective frameshift peptide rather than on a FUS truncation studied in isolation.

## Supplementary information

**Additional file 1 : Table S1**. Reported mutations in the *FUS* gene resulting in a frameshift tail. **Figure S1**. The effect of C-terminal truncations on stress granule recruitment of FUS protein. **Figure S2**. The effect of frameshift peptides on distribution and stability of GFP protein. Materials and methods

## Data Availability

The datasets used and/or analysed during the current study are available from corresponding authors on reasonable request.

## Abbreviations

ALS: Amyotrophic lateral sclerosis
CHX: Cycloheximide
FUS: Fused in sarcoma
NLS: Nuclear localisation signal
RGG: Arg-Gly-Gly-rich motif
RRM: RNA recognition motif
SG: Stress granule
