# Supplementary material for: Frameshift peptides alter the properties of truncated FUS proteins in ALS-FUS
Source: Mol Brain. 2020 May 13;13:77. doi: 10.1186/s13041-020-00618-0 (PMC7222445; doi:10.1186/s13041-020-00618-0)
Supplement: Supplementary file 1 — Additional file 1 : Table S1. Reported mutations in the FUS gene resulting in a frameshift tail. Figure S1. The effect of C-terminal truncations on stress granule recruitment of FUS protein. Figure S2. The effect of frameshift peptides on distribution and stability of GFP protein. Materials and methods [file 13041_2020_618_MOESM1_ESM.docx]

**Additional file 1**

**Frameshift peptides alter the properties of truncated FUS proteins in ALS-FUS**

Haiyan An, Camille Rabesahala de Meritens, Vladimir L. Buchman and Tatyana A. Shelkovnikova*

*Corresponding author e-mail: [shelkovnikovat@cardiff.ac.uk](mailto:shelkovnikovat@cardiff.ac.uk)

This file contains:

- Table S1. Reported mutations in the *FUS* gene resulting in a frameshift tail
- Figure S1. The effect of C-terminal truncations on stress granule recruitment of FUS protein
- Figure S2. The effect of frameshift peptides on distribution and stability of GFP protein.
- Materials and methods

**Table S1. Reported mutations in the *FUS* gene resulting in a frameshift peptide tail**

| Gene mutation | Mutant protein | Mutation type | Predicted C-terminal frameshift peptide sequence | Age of onset | Reference |
| --- | --- | --- | --- | --- | --- |
| g.10747A>G; IVS13-2A>G | p.G466VfsX14 | splicing mutation | VSTDRIAGRGRIN* | 20 | DeJesus-Hernandez, M., et al. (2010) |
| c.1432_1478del GGCTATGATCGAGGCGGCTACCGGGGCCGCGGCGGGGACCGTGGAGG | p.G478LfsX23 | deletion | LPRGPGWWGQRWLWPWQDGFQG* | 21, 26 | Waibel, S., et al. (2013) |
| c.1419_1420insGT | p.G474VfsX56 | insertion | VVAEEAMIEAATGAAAGTVEASEGAGVVGTEVALALARWIPGVSTDRIAGRGRIN* | 26 | Hara, M., et al. (2012) |
| c.1449_1488del  CTACCGGGGCCGCGGCGGGGACCGTGGAGGCTTCCGAGGG | p.Y484AfsX32 | deletion | AGVVGTEVALALARWIPGVSTDRIAGRGRIN* | N/A | Yan, J., et al. (2010) |
| c. 1475delG | p.G492EfsX37 | deletion | EASEGAGVVGTEVALALARWIPGVSTDRIAGRGRIN* | 17 | Yamashita, S., et al. (2012) |
| c.1483delC | p.R495EfsX34 | deletion | EGAGVVGTEVALALARWIPGVSTDRIAGRGRIN* | 23, 72 | Yan, J., et al. (2010) |
| c. 1484delG | p.R495QfsX34 | deletion | QGAGVVGTEVALALARWIPGVSTDRIAGRGRIN* | 19 | Belzil, V. V., et al. (2012) |
| c.1485delA | p.G497AfsX32 | deletion | AGVVGTEVALALARWIPGVSTDRIAGRGRIN* | 12.5 | Yan, J., et al. (2010) |
| c.1506dupA | p.D502EfsX15 | duplication | EQRWLWPWQDGFQG* | 49 | Belzil, V. V., et al. (2011) |
| c.1509_1510delAG | p.G504WfsX12 | deletion | WLWPWQDGFQG* | 15, 23, 33 | Kent, L., et al. (2014) |
| c.1527insTGGC | p.K510WfsX8 | insertion | WQDGFQG* | 23, 46 | Yan, J., et al. (2010) |
| c.1542-2A>C | p.G515SfsX8 | splicing mutation | SMSRSGR* | N/A | Belzil, V. V., et al. (2010) |
| c.1554_1557delACAG | p.Q519IfsX9 | deletion | IAGRGRIN* | 18 | [Bäumer](https://www.ncbi.nlm.nih.gov/pubmed/?term=B%26%23x000e4%3Bumer%20D%5BAuthor%5D&cauthor=true&cauthor_uid=20668261), D., et al. (2010) |

**common peptide sequences are given in the same colour*


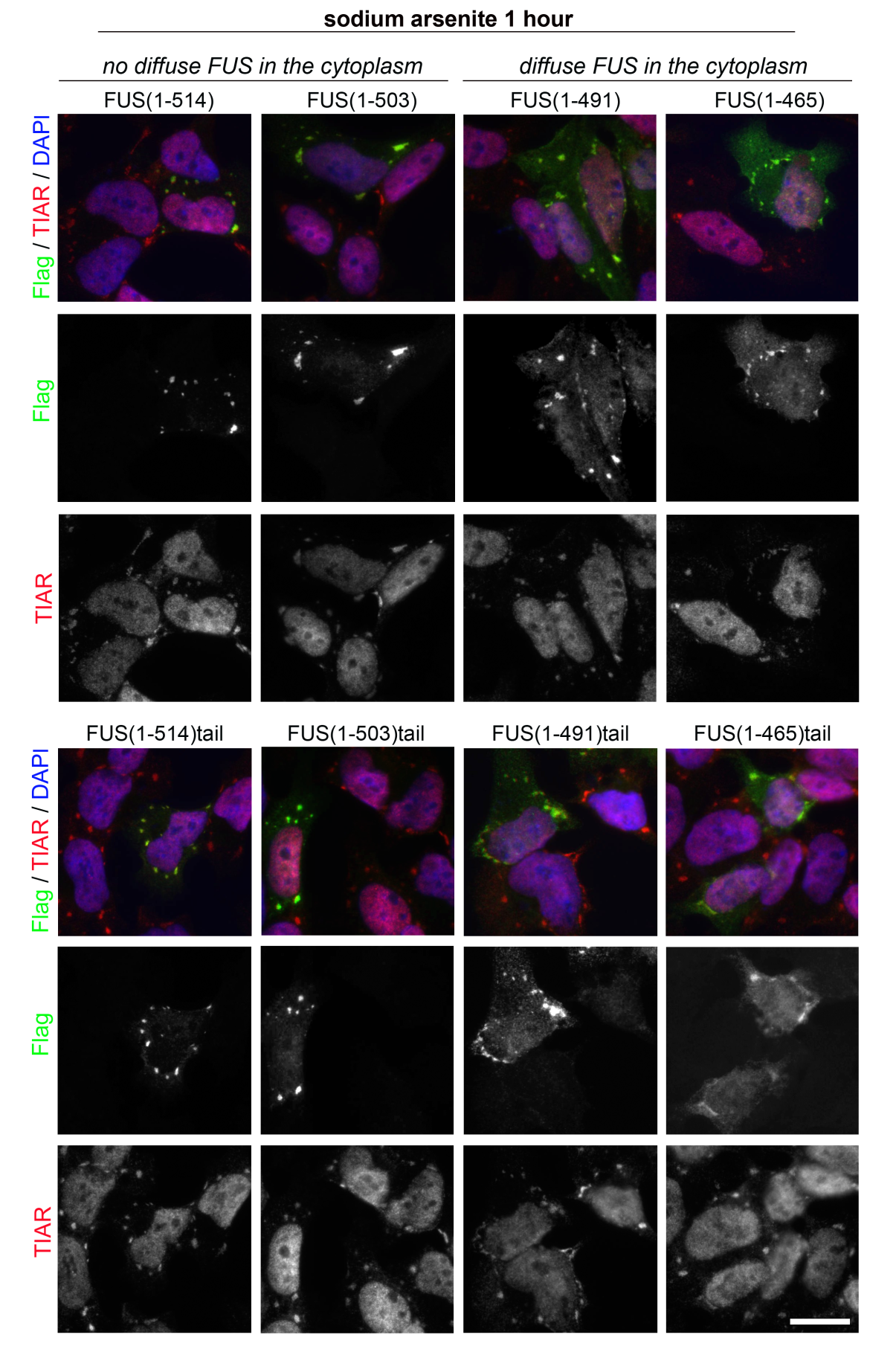


**Figure S1.** Longer truncations affecting RNA-binding domains of FUS diminish its affinity to SGs, and frameshift tails do not affect FUS sequestration into SGs during stress. Neuroblastoma cells were transfected to express FUS variants, and after 24 h subjected to sodium arsenite stress for 1 h. TIAR was used as a SG marker. Scale bar, 10 µm.

**
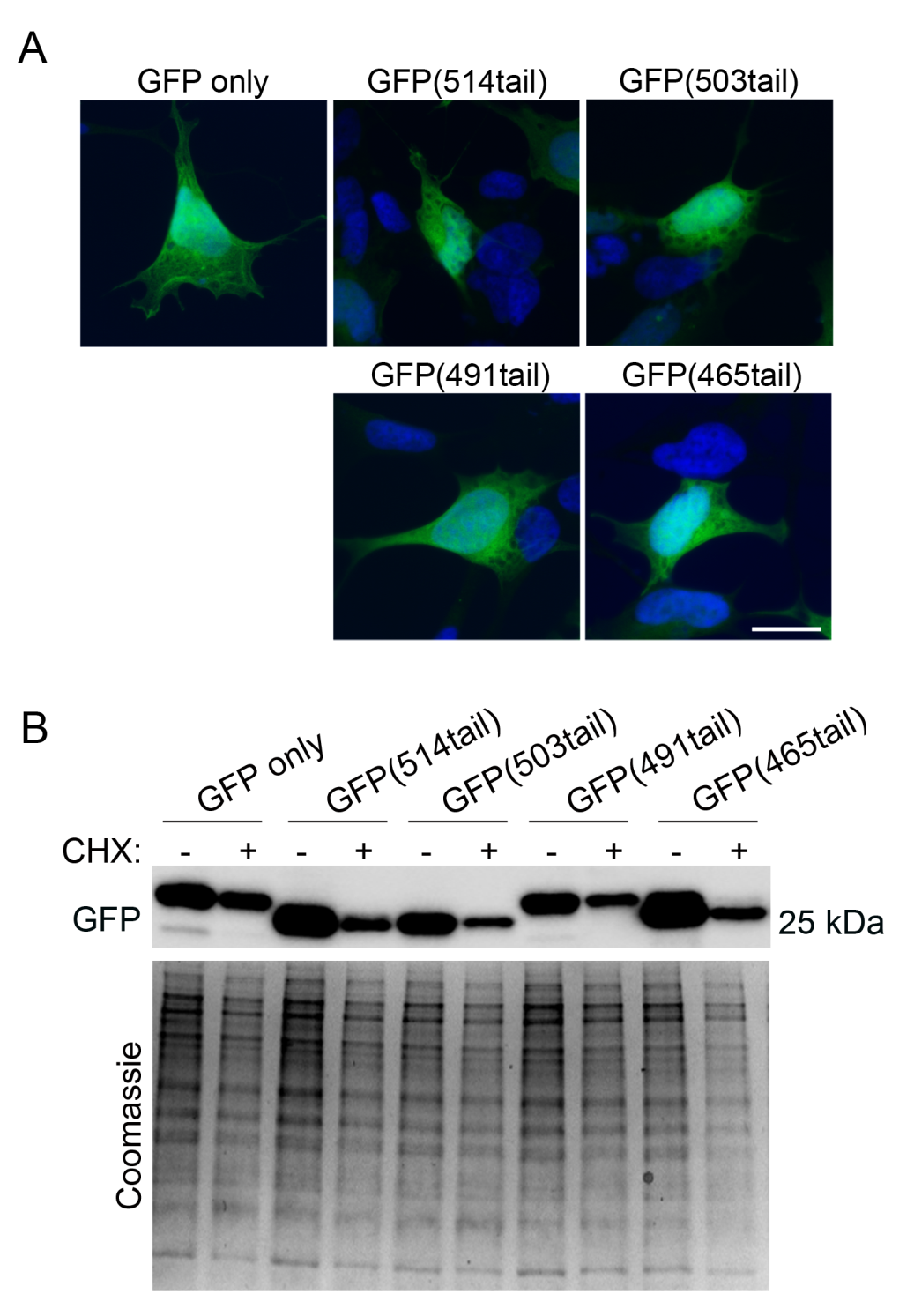
**

**Figure S2.** Attachment of mutant FUS frameshift peptide tails does not alter the distribution of GFP protein and does not increase its stability. (A) Neuroblastoma cells were transfected to express GFP only or GFP tagged with the indicated FUS frameshift tail and analysed after 24 h. Representative images are shown. Scale bar, 10 µm. (B) Neuroblastoma cells were transfected to express GFP only or GFP tagged with the indicated FUS frameshift tail, and 24 h post-transfection treated with cycloheximide (CHX) for 36 h. A representative western blot is shown.

**Materials and methods**

*Cloning*

pEGFP-C1 vector (Clontech) or pFLAG-CMV-4 vector (Sigma) were used for the construction of plasmids expressing N-terminally GFP- or Flag-tagged truncated FUS proteins with/without a peptide tail. Human WT cDNA was used to amplify the PCR fragments that encode FUS proteins with the desired peptide sequences using common forward FUS primer and a specific reverse primer. Plasmids for expression of WT FUS, FUS(1-465) and FUS(1-514) were generated in a previous study (Shelkovnikova et al., 2014). pEGFP-C1 vector was used to amplify the PCR fragments that encode GFP proteins with the desired peptide sequences using the common forward GFP primer and a specific reverse primer. The peptide-encoding sequence was embedded in the reverse primer:

| construct | Specific reverse primer |
| --- | --- |
| FUS(1-465)tail | 5’-CGGGATCCCTAATTAATACGGCCTCTCCCTGCGATCCTGTCTGTGCTCACCCCCATGTGAGAGCCACCTG-3’ |
| FUS(1-491) | 5’-CGGGATCCTTAACGGTCCCCGCCGCGGCCCCGGTAG-3’ |
| FUS(1-491)tail | 5’-CGGGATCCTTAATTAATACGGCCTCTCCCTGCGATCCTGTCTGTGCTCACCCCTGGAATCCATCTTGCCAGGGCCAAAGCCACCTCTGTCCCCACCACCCCACGGTCCCCG-3’ |
| FUS(1-503) | 5’-CGGGATCCTTATCTGTCCCCACCACCCCGGC-3’ |
| FUS(1-503)tail | 5’-CGGGATCCTTACCCCTGGAATCCATCTTGCCAGGGCCAAAGCCATCTGTCCCCACCACCCCGGC-3’ |
| FUS(1-514)tail | 5’-CGGGATCCTTACCTTCCTGATCGGGACATCGATCTGGAATCCATCTTGCCAG-3’ |
|  | **Common forward FUS primer** |
| All constructs | 5’-GCTAAAGCAGCTATTGACTG-3’ |
|  | **Common forward GFP primer** |
| All constructs | 5’-TTACGGTAAACTGCCCACTTG-3’ |

*Cell maintenance and transfection*

SH-SY5Y neuroblastoma cells were maintained in 1:1 mixture of Dulbecco’s Modified Eagle’s Medium (DMEM) and F12 medium supplemented with 10% foetal bovine serum (FBS), 50 U/ml penicillin-streptomycin, and 500 μM L-glutamine (all Invitrogen). Lipofectamine™ 2000 Transfection Reagent (Thermo Scientific) was used for transfection. Cells were treated with 0.5 mM sodium arsenite (Sigma) for 1 h.

*Immunofluorescent analysis*

Cells grown on coverslips were fixed with 4% cold paraformaldehyde for 15 min at room temperature and permeabilised with methanol. Cells were incubated with primary antibody diluted with PBS-T containing 5% goat serum at room temperature for 2 h or at 4 °C overnight. Fluorochrome-conjugated secondary antibodies (AlexaFluor, Life Technologies) diluted 1:1,000 in PBS-T were applied at room temperature for 1.5 h. Nuclei were stained with DAPI and mounted on glass slides using Immumount (Thermo Scientific). Fluorescent images were taken using BX57 fluorescent microscope equipped with ORCA-Flash 4.0 camera (Hamamatsu) and cellSens Dimension software (Olympus). Figures were prepared using Photoshop CS3 or PowerPoint 2016 software. The primary antibodies used for staining: anti-Flag (rabbit monoclonal, Cell Signaling), anti-TIAR (mouse monoclonal, BD Biosciences). Quantification of cells with cytoplasmic granules/clusters was performed in a blinded fashion. Nuclear-cytoplasmic distribution of FUS was quantified using Image J <https://imagej.nih.gov/ij/>

*Protein stability analysis and western blotting*

SH-SY5Y cells were transfected with equal amounts of corresponding plasmids and allowed to express proteins for 24 h. Cycloheximide (Sigma) was added directly to the media to a final concentration of 20 µg/ml to block translation; cells were harvested after 36 h of cycloheximide exposure. SDS-PAGE loading buffer was used to lyse cells directly on plates, followed by heating to 100ºC for 10 min. Proteins were resolved using hand-casted polyacrylamide gels (Bid-Rad), and transferred to PVDF membrane by semi-dry blotting followed by blocking, incubation with primary and HRP-conjugated secondary (GE Healthcare) antibodies and ECL detection. The following commercial antibodies were used for western blotting: GFP (mouse monoclonal, Santa Cruz); beta-actin (mouse monoclonal, clone AC15, Sigma). Quantification of protein levels was performed using the respective tool of Image J.

*Statistics*

Statistical analysis was performed using GraphPad Prism 6.0 software. Results were considered statistically significant at p < 0.05.

**References**

*Baumer D, Hilton D, Paine SM, Turner MR, Lowe J, Talbot K, Ansorge O:* ***Juvenile ALS with basophilic inclusions is a FUS proteinopathy with FUS mutations****. Neurology 2010,* ***75****(7):611-618.*

*Belzil VV, Daoud H, St-Onge J, Desjarlais A, Bouchard JP, Dupre N, Lacomblez L, Salachas F, Pradat PF, Meininger V et al:* ***Identification of novel FUS mutations in sporadic cases of amyotrophic lateral sclerosis****. Amyotroph Lateral Scler 2011,* ***12****(2):113-117.*

*DeJesus-Hernandez M, Kocerha J, Finch N, Crook R, Baker M, Desaro P, Johnston A, Rutherford N, Wojtas A, Kennelly K et al:* ***De novo truncating FUS gene mutation as a cause of sporadic amyotrophic lateral sclerosis****. Hum Mutat 2010,* ***31****(5):E1377-1389.*

*Hara M, Minami M, Kamei S, Suzuki N, Kato M, Aoki M:* ***Lower motor neuron disease caused by a novel FUS/TLS gene frameshift mutation****. J Neurol 2012,* ***259****(10):2237-2239.*

*Kent L, Vizard TN, Smith BN, Topp SD, Vance C, Gkazi A, Miller J, Shaw CE, Talbot K:* ***Autosomal dominant inheritance of rapidly progressive amyotrophic lateral sclerosis due to a truncation mutation in the fused in sarcoma (FUS) gene****. Amyotroph Lateral Scler Frontotemporal Degener 2014,* ***15****(7-8):557-562.*

*Shelkovnikova TA, Robinson HK, Southcombe JA, Ninkina N, Buchman VL:* ***Multistep process of FUS aggregation in the cell cytoplasm involves RNA-dependent and RNA-independent mechanisms****. Hum Mol Genet 2014,* ***23****(19):5211-5226.*

*Waibel S, Neumann M, Rosenbohm A, Birve A, Volk AE, Weishaupt JH, Meyer T, Muller U, Andersen PM, Ludolph AC:* ***Truncating mutations in FUS/TLS give rise to a more aggressive ALS-phenotype than missense mutations: a clinico-genetic study in Germany****. Eur J Neurol 2013,* ***20****(3):540-546.*

*Yamashita S, Mori A, Sakaguchi H, Suga T, Ishihara D, Ueda A, Yamashita T, Maeda Y, Uchino M, Hirano T:* ***Sporadic juvenile amyotrophic lateral sclerosis caused by mutant FUS/TLS: possible association of mental retardation with this mutation****. J Neurol 2012,* ***259****(6):1039-1044.*

*Yan J, Deng HX, Siddique N, Fecto F, Chen W, Yang Y, Liu E, Donkervoort S, Zheng JG, Shi Y et al:* ***Frameshift and novel mutations in FUS in familial amyotrophic lateral sclerosis and ALS/dementia****. Neurology 2010,* ***75****(9):807-814.*
